# Omega-3 nanoemulgel in prevention of radiation-induced oral mucositis and its associated effect on microbiome: a randomized clinical trial

**DOI:** 10.1186/s12903-023-03276-5

**Published:** 2023-08-30

**Authors:** Basma M. Morsy, Shahira El Domiaty, Mohamed A. M. Meheissen, Lamia A. Heikal, Marwa A. Meheissen, Nourhan M. Aly

**Affiliations:** 1 Oral Medicine, Periodontology, Oral Diagnosis, and Oral Radiology Department, Faculty of Dentistry, Alexandria University, Champolion St, 21527 Alexandria Governorate, Egypt; 2 Clinical Oncology and Nuclear Medicine Department, Faculty of Medicine, Alexandria University, Alexandria Governorate, Egypt; 3 Department of Pharmaceutics, Faculty of Pharmacy, Alexandria University, Alexandria Governorate, Egypt; 4 Medical Microbiology and Immunology Department, Faculty of Medicine, Alexandria University, Alexandria Governorate, Egypt; 5 Pediatric Dentistry and Dental Public Health Department, Faculty of Dentistry, Alexandria University, Alexandria Governorate, Egypt

**Keywords:** Oral mucositis, Prevention, Omega-3, Nanoemulgel, Microbiome

## Abstract

**Background:**

Oral mucositis (OM) is recognized as one of the most frequent debilitating sequelae encountered by head and neck cancer (HNC) patients treated by radiotherapy. This results in severe mucosal tissue inflammation and oral ulcerations that interfere with patient’s nutrition, quality of life (QoL) and survival. Omega-3 (ω-3) polyunsaturated fatty acids (PUFAs) have recently gained special interest in dealing with oral diseases owing to its anti-inflammatory, anti-oxidant and wound healing properties. Thus, this study aims to assess topical Omega-3 nanoemulgel efficacy in prevention of radiation-induced oral mucositis and regulation of oral microbial dysbiosis.

**Materials and methods:**

Thirty-four head and neck cancer patients planned to receive radiotherapy were randomly allocated into two groups: Group I: conventional preventive treatment and Group II: topical Omega-3 nanoemulgel. Patients were evaluated at baseline, three and six weeks after treatment using the World Health Organization (WHO) grading system for oral mucositis severity, Visual Analogue Scale (VAS) for perceived pain severity, and MD-Anderson Symptom Inventory for Head and Neck cancer (MDASI-HN) for QoL. Oral swabs were collected to assess oral microbiome changes.

**Results:**

VAS scores and WHO mucositis grades were significantly lower after six weeks of treatment with topical Omega-3 nanoemulgel when compared to the conventional treatment. The total MDASI score was significantly higher in the control group after three weeks of treatment, and the head and neck subscale differed significantly at both three and six weeks. A significant reduction in Firmicutes/Bacteroidetes ratio was observed after six weeks in the test group indicating less microbial dysbiosis.

**Conclusions:**

Topical Omega-3 nanoemulgel demonstrated a beneficial effect in prevention of radiation-induced oral mucositis with a possibility of regulating oral microbial dysbiosis.

## Background

Oral mucositis (OM) is considered one of the most common debilitating complications that develops in head and neck cancer (HNC) patients receiving radiotherapy [1].

Radiation-induced oral mucositis (RIOM) is described as an injury to normal tissues occurring in the form of oral mucosal inflammation and/or ulceration, encountered in more than 80% of head and neck cancer patients undergoing radiation therapy [2–4].

OM- related complaints frequently include mild to severe pain as well as oral erosions and ulcerations that are prone to secondary infections, which interfere with the oral intake of solid food and liquids and may subsequently result in dehydration and/or malnutrition. It can also lead to a reduction in the treatment dose or breaking the treatment course, prolonging hospitalization and consequently affecting patients’ quality of life (QoL) and overall survival [1, 3, 5].

The major steps in OM development and resolution are generally characterized by five continuous overlapping phases [1, 6]. This usually involves generation of reactive oxygen species (ROS), activation of transcription factors like nuclear factor kappa B (NF-κB) and upregulation of some pro-inflammatory cytokines including tumor necrosis factor alpha (TNF-α), interleukin-1 beta (IL-1β) and interleukin6 (IL-6) [6–8]. This further strengthens tissue injury resulting in development of painful ulcerations that are prone to secondary infection, which is considered the most significant stage in mucositis [7]. After 4–12 weeks of anti-cancer treatment completion, spontaneous healing of the oral tissues takes place, following submucosal signaling that promotes proliferation, differentiation and renewal of epithelial cells [9].

Along with the current cascade involved in the pathogenesis of OM, recent studies have discussed an emerging role for oral microbial community alterations in OM development and progression. This is possibly explained by an alteration in oral microbial diversity or an increase in pathogenic bacterial abundance during radiation, which is known as “dysbiosis”[10]. A recent review has reported a notably higher bacterial load in OM epithelial ulcerations, which postulated a potential correlation between certain microbial communities and OM severity [11]. More intriguingly, the oral microbiome profile can be used to anticipate and subsequently prevent the onset of severe RIOM [10]. However, the exact role of oral microbiome in OM pathogenesis remains unclear [1, 3, 12]. Thus, in spite of using different antimicrobial agents that target bacteria associated with severe forms of OM, none of the conducted clinical trials have achieved tangible promising outcomes [11, 13].

To date, standard treatment for OM usually focuses on pain control and rehydration [14]. However, multiple preventive methods have been tested in previous clinical studies, with possible potential in mitigating OM. These interventions include: anti-inflammatory medications, herbal medicine, growth factors, cytokines and photobiomodulation therapy [15, 16]. Several studies also recommend patients’ dietary modifications such as decreasing sugar intake, in addition to using calcium phosphate rinses and benzydamine mouthwash, to prevent RIOM development [17, 18]. In spite of the continuously evolving treatment and preventive approaches, definitive cost-effective treatment or preventive therapies for oral mucositis are still lacking [1, 3, 19].

Omega-3 (ω-3) polyunsaturated fatty acids (PUFAs) are classified as essential fatty acids that have long been recognized in normal growth, health, and disease risk reduction [20, 21]. They comprise a group of fatty acids, among which eicosapentaenoic acid (EPA, C20:5) and docosahexaenoic acid (DHA, C22:6) are the two vital bioactive forms in humans [20, 22]. Although EPA and DHA can be synthesized from the precursor Alpha-linolenic acid (ALA), this requires complex chemical reactions making their conversion less efficient in humans. Thus, consumption of food such as fish, fish oils or nutritional supplements rich in these two principal fatty acids is recommended [22].

Supported by both animal and human studies, mounting evidence has shown that ω-3 PUFAs, mainly EPA and DHA, have beneficial therapeutic roles in various human diseases such as diabetes, cardiovascular diseases and autoimmune conditions [21, 23].Moreover, growing research indicates that dietary intake of ω-3 PUFAs in cancer patients exhibits anti-neoplastic roles, enhances the efficacy of radiation and lowers cancer incidence and mortality rates [24]. Additionally, multiple studies have highlighted the effect of Omega-3 supplements’ consumption in maintaining body weight/composition and improving (QoL) of cancer patients [25, 26]. This is possibly attributed to their anti-inflammatory and anti-oxidant effects, and also their role in maintaining epithelial integrity and promoting whole-body wound healing [27, 28].

Several hypotheses have been postulated to explain the anti-inflammatory effect of Omega-3. It majorly focused on their capability in inhibiting nuclear factor kappa B (NF-κB) protein expression [27]. This comes along with downregulation of multiple pro-inflammatory cytokines including (TNFα), (IL-1β) and (IL-6) [27, 29]. Omega-3 also possess the ability to produce certain metabolites known as specialized pro-resolving mediators (SPMs) that include: resolvins D (RvD) and E (RvE) series and protectins that act as potent anti-inflammatory agents [30]. Furthermore, DHA was noted to induce upregulation of detoxification and antioxidant genes, reducing oxidative stress [27, 31].

Moreover, recent studies suggest the potential role of ω-3 in managing chemotherapy- or radiotherapy-related intestinal microbial dysbiosis, by upregulating beneficial bacteria with anti-inflammatory impact, that in turn modulates systemic inflammatory and immune responses of the host. In addition to its ability to decrease proportions of pathogenic microorganisms, which may collectively influence OM in terms of development, severity or healing [11, 27, 29]. However, the effect of ω-3 on oral microbiome is still not fully covered.

Compared to systemic supplements, animal studies have demonstrated a better effect of locally delivered ω-3 PUFAs in terms of decreasing inflammation, enhancing re-epithelialization and wound healing [28, 32, 33].

Both human and animal studies demonstrated the effect of ω-3 PUFAs on different oral diseases such as gingivitis, periodontal diseases and recurrent aphthous stomatitis (RAS) [34–39]. A recent clinical trial suggested that ω-3 PUFAs may exert a therapeutic potential against chemotherapy-induced mucositis [40]. To the best of our knowledge however, the effect of ω-3 PUFAs has not been investigated on (RIOM) yet. Thus, the purpose of this study was to evaluate the effect of topical Omega-3 nanoemulgel in prevention of radiation-induced oral mucositis and its associated pain, improvement of patients’ (QoL), as well as testing its effect on oral microbiome.

## Materials and methods

### Study design

A two-arm parallel randomized, controlled clinical trial was conducted on thirty-four head and neck cancer patients of both genders who were planned to receive radiotherapy from February to September 2022. Patients were selected from the outpatient clinic of Department of Clinical Oncology, Faculty of Medicine, Alexandria University. Prior to the study, patients were given a detailed explanation of the study and signed an informed consent according to the guidelines of the Ethical Committee of the Faculty of Dentistry, Alexandria University. The study was completed according to the principles of the modified Helsinki’s code for human clinical studies (2013) and CONSORT 2010 guidelines for reporting randomized clinical trials. It was also approved by the Research Ethics committee of the Faculty of Dentistry, Alexandria University (IRB NO: 00010556-IORG0008839- 0290-09/2021) in September 2021, and has been registered at ClinicalTrials.gov on 28/01/2022, registration number: (NCT05214495), [41]. Detailed trial protocol can be accessed and provided when requested.

### Participants

Participants were eligible if they had head and neck cancer proven malignancy and were planned to receive radiotherapy (50 Gy or above) using machine (Elekta unity linear accelerator, Sweden) [42] either as postoperative (adjuvant) or definitive therapy, aged above 18 from both sexes [43], and had good to moderate oral hygiene levels (simplified oral hygiene index scores ≤ 3) [44]. Patients were excluded if they were planned to receive concomitant chemotherapy or were diagnosed with any current oral viral/fungal infections. Patients under anticoagulants such as warfarin, heparin, or aspirin, suffering from any uncontrolled systemic diseases (such as diabetes, cardiovascular, liver disorder, renal dysfunction) or having interfering physical or intellectual disabilities that can affect the procedure were also excluded [2].

Before enrollment in the study, thorough medical and dental history was taken from all patients. All participants received phase one therapy that included: scaling and root planning (SRP), obturation of caries, removal of all septic foci detected intraorally and basic oral hygiene instructions’ demonstration [45]. Also, a brief introduction about the study and its objectives was given to all patients in their native language and a written informed consent was signed by them, that stated all possible outcomes, side effects of the treatment, as well as their right to withdraw at any time from the study.

### Sample size estimation

Sample size was estimated assuming 5% alpha error and 80% study power. Bakr et al. [2] reported no signs of oral mucositis (grade 0) after 6 weeks of topical oral vitamin oral D application in 60% of patients compared to 13.3% with conventional treatment. Topical omega-3 oral gel is assumed to have a similar effect as vitamin D [35, 46]. Based on comparison of proportions, sample size was calculated to be 16 patients per group, increased to 18 to make up for loss to follow-up. The total sample size required = number of groups × number per group = 2 × 18 = 36 patients. Sample size was calculated using G*Power (Version 3.1.9.4) [47].

### Randomization, blinding and allocation concealment

Randomization was performed using a computer-generated random allocation software [48]. Participants were allocated in blocks of four to one of the two study groups, using permuted block technique. Allocation numbers were sealed in opaque envelopes, and an assistant, who was not involved in the study, performed the treatment allocation. Blinding of participants and the main operator was challenging since several therapeutic agents with different regimens and doses were applied in the control group compared to the test group that only received topical Omega-3 nanoemulgel. Accordingly, a single placebo gel for the control group was not feasible. However, the outcome assessor and statistician were blinded to the allocation of groups. Outcome assessment was performed by a trained oral medicine specialist after calibration using 20 intraoral photographs of different grades of mucositis. Intra-examiner reliability was calculated, and Kappa statistic ranged from 0.83 to 0.94 indicating excellent reliability [49].

### Preparation of Omega-3 nanoemulgel

Fish oil (Omega-3 fatty acids 70.4%; EPA 34.9% and DHA 24.2%) was supplied from Safe pharmaceutical company, Alexandria, Egypt. To enhance fish oil ingredients’ absorption and tissue-penetration, Omega-3 nanoemulgel was formulated [50]. Fish oil nano emulsion was developed using direct emulsification method. Briefly, the aqueous phase containing Tween 80 (El Gomhouria Co. Alexandria, Egypt) was added to the oily phase (Fish oil and Span 80 (sigma Aldrich, UK)) [51, 52]. The ratio of water: oil was 6:4 and the ratio of the used surfactant was 7:3 for Tween 80 and Span 80 respectively. The mixture was pre-emulsified for 5 min at 20,000 rpm using T25-digital Ultra-Turrax homogenizer (IKA Works, Inc., Wilmington, NC). The formed coarse emulsion was then ultrasonicated using Branson Digital Sonifier S-450D (Emerson Electric Co., St. Louis, MO at 60% ultrasonic amplitude for 5 min [53]. Nanoemulgel was prepared by mixing the nanoemulsion (NE) with Carbopol gel (1% w/w) in 2: 1 ratio. For preparation of gel, Carbopol 940 (Alamreya Pharmaceuticals, Alexandria, Egypt) was dispersed in water then neutralized using triethanolamine using pH meter (Mettler Toledo, Switzerland) [54]. Drops of Apple oil were added at the end as a flavouring agent. The final concentration of Fish oil in the preparation was 35% w/w.

### Invitro characterization of the prepared formulation

1. **Particle Size Distribution Analysis** Size of the emulsion droplets and polydispersity index (PDI) was measured using a Zetasizer Nano ZS90 (Malvern Pananalytical, Malvern, UK). Samples were diluted 1:10 using distilled water. Dynamic Light Scattering was performed using laser wavelength 633 nm and 90° scattering angle at 25 °C.
2. **Viscosity measurement**: The viscosity of the prepared (NE) hydrogel was measured using Brookfield RV head multipoint viscometer at a speed of 1 rpm and spindle CP-40 at room temperature.
3. **Spreadability**: The spreadability of prepared nanoemulgel was determined by measuring the spreading diameter of nanoemulgel between the two glass plates after 1 min. The spreadability was calculated using the formula 𝑆 = 𝑚⋅𝑙 /𝑡, where S is spreadability, m is mass added, l diameter of the spreaded gel and t is the time taken.
4. **Stability Test**: The stability of the prepared (NE) systems was assessed by applying centrifugation stress; 1 mL of the system was added to 100 mL distilled water and centrifuged for 30 min (5000× rpm), and phase separation was inspected visually using ultra cooling centrifuge (Sigma laborzentrifugen GmbH, Osterode, Germany) [51].
5. **Characterization Results**: Characterization results for Omega-3 nanoemulgel are listed in Table 1.

**Table 1:** Characterization results for Omega-3 nanoemulgel

Characteristics of NE	
Droplet size of NE (nm)	146.7 ± 11.45
PDI	0.14
Stability	Stable with no phase separation
Characteristics of FO nano emulgel	
Viscosity	18,260 cp.
Spreadability	0.79 g.cm.S^− 1^
pH	6.8

### Intervention

The assigned thirty-four patients were randomly allocated to the following groups:

The control group (n = 17) received conventional preventive treatment that was started one day before radiotherapy and applied twice daily for six weeks [2, 55]. This included topical antifungal agent: Miconazole 2% (Miconaz oral gel)^1^ that was applied with a standardized spoon twice daily, anti-inflammatory mouthwash, 5gm sodium bicarbonate mouthwash (Alkamisr sachets)^2^ where patients were asked to rinse twice daily using a standardized cup. Topical anesthetics agent containing 1.5 g Benzocaine: (BBC oral spray)^3^, topical analgesic gel containing 2.0 g Lidocaine HCl (Oracure gel)^4^ and systemic analgesics were provided when needed.

The test group (n = 17) were given topical Omega-3 nanoemulgel formulated and characterized with the aid of the Pharmaceutics Department, Faculty of Pharmacy, Alexandria University, as previously mentioned, where 1 g (containing 350 mg of fish oil) was self-applied by patients twice daily, every 12 h, starting one day before radiotherapy and ongoing for six weeks, to ensure a daily dose of (700 mg of fish oil/day) [56–58]. To ensure compliance, participants of both groups were given self-check reminder sheets with assigned doses, that were checked at both follow up visits. Also, patients developing grade (3–4) OM from both groups were re-assessed by an oncology specialist. Systemic analgesics, parenteral nutrition or complete hospitalization was done if needed.

### Outcome measures

As patients were planned to receive a typical radiation cycle of (5–7) weeks, and as OM clinical signs usually appear by the second or third week of treatment [59], all patients were evaluated clinically at baseline, three and six weeks after the intervention [2, 8] through the following:

- Oral mucositis severity, measured using the WHO grading system [60], according to the patient’s functional and symptomatic clinical features [61] listed in Table 2.
- Pain and discomfort were reported by each patient using the Visual Analog Scale (VAS) [62, 63] where patients were asked to rate the level of pain they were experiencing on a scale from 0 (no pain at all) to 10 (severe intolerable pain).
- Symptom burden and QoL were assessed by the translated Arabic version of M. D. Anderson Symptom Inventory–Head and Neck (MDASI-HN) questionnaire [64, 65]. It assesses the severity of symptoms experienced by head and neck cancer patients that interfere with their daily functioning. The MDASI-HN is composed of 28 items measured on 3 subscales: 13 core symptom items (as: fatigue, nausea and dry mouth), 6 interference items (as activity and life enjoyment), and 9 head and neck cancer specific items (as difficulty in swallowing/chewing and mouth or throat sores). Each item is scored on a Likert scale from 0 to 10 with higher scores indicating a worse condition. The score of each domain is the average score of its items, and the total score is the average of the 28 items. The questionnaire was originally developed and validated in English [64], and further translated and validated in Arabic [65]. The scores were used as quantitative variables.

**Table 2:** WHO grading system for Oral mucositis severity

Grade	Description
Grade 0	None
Grade I	Mild mucositis: oral soreness and/or erythema
Grade II	moderate mucositis: oral erythema, ulcers, and solid diet can be tolerated
Grade III	severe mucositis: oral ulcers, but solid diet cannot be tolerated
Grade IV	life-threatening: oral alimentation is impossible

### Microbiological assessment

#### Sample collection, preservation, and transport

Oral samples were collected from all patients using sterile cotton tipped swabs which were used to remove saliva and shedded cells by gently scrapping the oral mucosa. Swabs were then put into 3 ml sterile phosphate buffered saline (PBS) and were immediately delivered and stored at − 20 °C, at Alexandria University Diagnostic Microbiology Laboratory.

#### DNA extraction

DNA was extracted from samples using QIAamp® DNA Mini Kit (QIAGEN, Hilden, Germany) according to the manufacturers’ instructions. DNA extracts were stored at − 80 °C until PCR testing. Eight microliters of DNA extract were used for each PCR reaction.

#### Real-time PCR (SYBR green)

The real-time PCR protocol was done as previously described [66]. Selected phyla; (Bacteroidetes [67], Firmicutes [68]) were targeted using specific PCR primers. In addition to a broad-range primer which targets conserved 16SrRNA sequence of total bacteria [69], the amplification of which acted as the denominator against which other bacteria amplification was estimated. Primers (Metabion International AG, Germany) that were used in the study are listed in Table 3. Amplification was carried out in real-time PCR cycler, the Rotor-Gene Q (QIAGEN, Germany) by the aid of 2x MAXIMA SYBR Green qPCR Master mix (2x), No-ROX (Thermo Fisher Scientific). It was performed in 20 µL reaction volume that contains 10 pmol/µL of each primer. The reaction consisted of initial denaturation at 95 °C for 10 min, followed by 40 cycles of denaturation at 95 °C for 30 s, annealing at 60 °C for 30 s, and extension at 72 °C for 30 s. Melting curve analysis was done to check the specificity of the amplified products. Specific bacterial DNA quantification was expressed as relative quantitation/ fold difference that was calculated automatically using the Rotor-Gene software [70].

**Table 3:** Primers’ sequences used in the current study

GENE	SEQUENCE	bp
**Total bacteria**	CGCTAGTAATCGTGGATCAGAATG TGTGACGGGCGGTGTGTA	69
**Phylum**		
Bacteroidetes	GTTTAATTCGATGATACGCGAG TTAASCCGACACCTCACGG	122
Firmicutes	GGAGYATGTGGTTTAATTCGAAGCA AGCTGACGACAACCATGCAC	126

### Statistical analysis

Data were analyzed using IBM SPSS for Windows (Version 23.0) and significance was inferred at p value < 0.05. Descriptive statistics were calculated as means, standard deviation (SD), median, interquartile range (IQR), frequencies and percentages. Comparisons between the two study groups were done using independent samples t-test for quantitative normally distributed variables, and Mann-Whitney test for non-normally distributed and ordinal. Fisher exact and chi-square tests were used for comparing qualitative nominal variables. Comparisons between different timepoints within the same group were done using Wilcoxon signed rank (two time points only), and Friedman test (3 timepoints) followed by multiple pairwise comparisons using Bonferroni adjusted significance level. Intention-to-treat analysis was used in analyzing all subjects included in the current study.

## Results

**Fig. 1:**
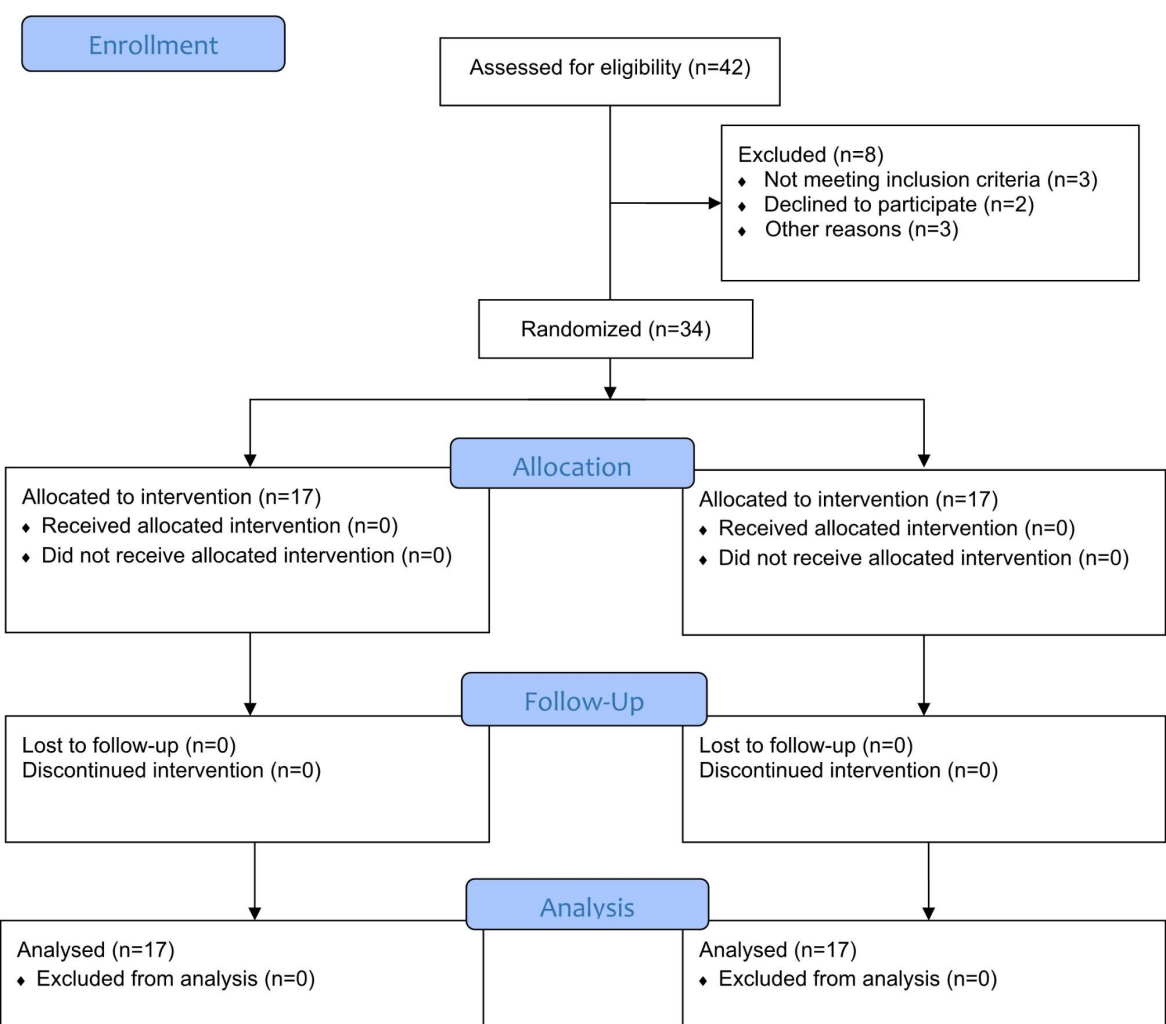
Consort flow chart

Figure 1 shows that out of the total 42 head and neck cancer patients assessed for eligibility, only 34 were included in the current study. Table 4 represents the patients’ demographic data and baseline characteristics. There were 9 (52.9%) males in the test group compared to 13 (76.5%) in the control group. The mean (SD) age was 55.76 (11.67) and 58.35 (11.67) in the test and control groups, respectively. The most common cancer site was the oral cavity in both groups. No statistically significant differences between groups were detected regarding their demographic data and baseline characteristics. The only complaint received was a transient bitter taste associated with topical Omega-3, and no other adverse side-effects were observed in both groups.

**Table 4:** Baseline characteristics

		Test (n = 17)	Control (n = 17)	P value
**Age a**	**Mean (SD)**	55.76 (11.67)	58.35 (11.67)	0.52^a^
**Gender: n (%)**	**Males**	9 (52.9%)	13 (76.5%)	0.28^b^
	**Females**	8 (47.1%)	4 (23.5%)	
**Diagnosis: n (%)**	**Laryngeal**	5 (29.4%)	6 (35.3%)	0.81^c^
	**Oral Cavity**	9 (52.9%)	9 (52.9%)	
	**Sinonasal**	3 (17.7%)	2 (11.8%)	
**Radiation therapy: n (%)**	**Adjuvant**	14 (82.3%)	10 (58.8%)	0.26^b^
	**Definitive**	3 (17.7%)	7 (41.2%)	
**Total radiation dose (GY)**	**Mean (SD)**	64.20 (3.78)	65.18 (4.55)	0.52^a^
	**Median (IQR)**	64.0 (60.0, 66.0)	65.5 (60.0, 70.0)	
	**Min – Max**	60.0–70.0	60.0–70.0	
**Number of radiation fractions**	**Mean (SD)**	31.87 (2.17)	32.18 (2.65)	0.72^a^
	**Median (IQR)**	32.0 (30.0, 33.0)	32.0 (30.0, 35.0)	
	**Min – Max**	28.0–35.0	28.0–35.0	

^a^: Independent samples t-test

^b^: Fisher exact test

^c^: Chi-square test (with Monte Carlo corrected p value)

**Fig. 2:**
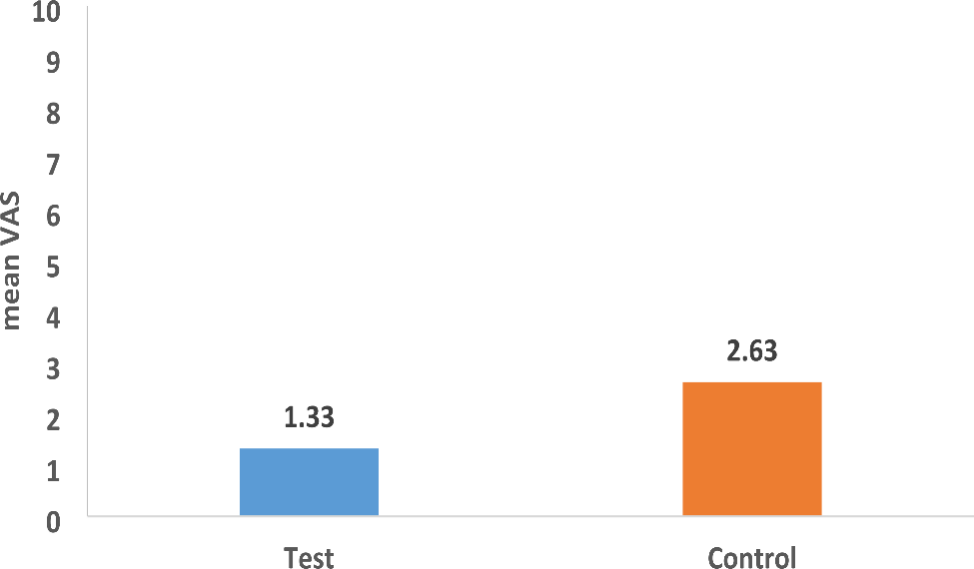
Difference in mean VAS score after 6 weeks (from baseline)

Table 5 shows that there were no significant differences in the perceived pain intensity between the two groups at baseline (p = 0.56) and three weeks (p = 0.09), while at six weeks, the control group showed significantly higher perceived pain than the test group (mean (SD) = 2.75 (2.21) and 1.40 (1.55) in the control and test groups, respectively, p = 0.049). The mean difference in pain intensity after six weeks (from baseline) was higher in the control group (mean (SD) = 2.63 (2.28)) than the test group (mean (SD) = 1.33 (1.63)), (p = 0.09) as represented in Fig. 2.

**Table 5:** Comparison of pain intensity using VAS in the two study groups at different timepoints

		Test (n = 17)	Control (n = 17)	MWU P value
**Baseline**	**Mean (SD)**	0.06 (0.24)	0.18 (0.39)	0.56
	**Median (IQR)**	0.00 (0.00, 0.00)	0.00 (0.00, 0.00)	
**3 weeks**	**Mean (SD)**	1.79 (2.42)	3.06 (2.46)	0.09
	**Median (IQR)**	1.00 (0.00, 3.25)	3.00 (1.00, 5.00)	
**6 weeks**	**Mean (SD)**	1.40 (1.55)	2.75 (2.21)	0.049*
	**Median (IQR)**	1.00 (0.00, 2.00)	2.00 (1.00, 4.75)	
**Friedman test p value**		0.06	< 0.001*	
**Baseline vs. 3 weeks**		0.17	< 0.001*	
**Baseline vs. 6 weeks**		0.07	0.002*	
**3 weeks vs. 6 weeks**		1.00	1.00	

MWU: Mann-Whiney U test was used

*statistically significant at p value < 0.05

Table 6 shows that all the included patients presented with grade 0 mucositis at baseline. After three weeks of therapy, 10 patients (58.8%) in the test group compared to only 3 patients (17.6%) in the control group showed grade 0 mucositis, while severe mucositis (grade 3–4) was reported in only three patients (17.7%) in the test group compared to 7 (41.2%) in the control group with a significant difference between both groups (p = 0.01). At the six weeks follow-up, the same 10 patients (58.8%) presented with grade 0 mucositis in the test group, compared to only 2 patients (11.8%) in the control group with a significant difference between both groups (p = 0.02) highlighting the preventive activity of Omega-3 nanoemulgel. Moderate mucositis (grade 1–2) was reported in 11 patients (64.7%) in the control group compared to 5 patients (29.4%) in the test group, and severe mucositis (grade 3–4) was reported in 2 (11.8%), and 4 (23.5%) in the test and control groups, respectively. As for the intragroup comparisons, there were no significant differences between the mucositis scores across timepoints in the test group (p = 0.08), however, significant differences were detected between baseline and both three and six weeks follow-ups in the control group (p < 0.001 and 0.002, respectively).

**Table 6:** Comparison of WHO oral mucositis grade between the two study groups at different timepoints

		Test (n = 17)	Control (n = 17)	P value
**Baseline**	**Grade 0**	17 (100%)	17 (100%)	1.00
	**Grade 1–2**	0 (0%)	0 (0%)	
	**Grade 3–4**	0 (0%)	0 (0%)	
**3 weeks**	**Grade 0**	10 (58.8%)	3 (17.6%)	0.01*
	**Grade 1–2**	4 (23.5%)	7 (41.2%)	
	**Grade 3–4**	3 (17.7%)	7 (41.2%)	
**6 weeks**	**Grade 0**	11 (64.7%)	2 (11.8%)	0.02*
	**Grade 1–2**	4 (23.5%)	11 (64.7%)	
	**Grade 3–4**	2 (11.8%)	4 (23.5%)	
**Friedman’s test**		0.08	< 0.001*	
**Baseline vs. 3 weeks**		0.13	< 0.001*	
**Baseline vs. 6 weeks**		0.36	0.002*	
**3 weeks vs. 6 weeks**		1.00	1.00	

*statistically significant at p value < 0.05

Table 7 shows that the total MDASI scores did not differ significantly between the two study groups at baseline and six weeks (p = 0.79 and 0.16, respectively). At three weeks, the total MDASI score was significantly higher in the control than the test group (mean (SD) = 3.04 (1.23) and 2.34 (1.43), in the control and test groups, respectively). As for the Head and Neck subscale, scores were significantly higher in the control group at three and six weeks (p = 0.04 and 0.02, respectively), while in the interference subscale the significant difference between both groups was only detected at the three weeks follow-up (p = 0.04).

**Table 7:** Comparison of MDASI scores between the two study groups at different timepoints

		Test (n = 17)	Control (n = 17)	P value
		Mean (SD)		
**Core**	**Baseline**	0.6 (0.40)	0.43 (0.31)	0.10
	**3 weeks**	2.09 (1.85)	2.44 (1.31)	0.28
	**6 weeks**	1.94 (0.75)	2.40 (1.26)	0.28
	**Friedman test**	< 0.001*	< 0.001*	
	**Baseline vs. 3 weeks**	0.003*	< 0.001*	
	**Baseline vs. 6 weeks**	< 0.001*	< 0.001*	
	**3 weeks vs. 6 weeks**	1.00	1.00	
**Interference**	**Baseline**	1.23 (0.81)	1.24 (1.01)	0.92
	**3 weeks**	2.56 (1.59)	3.28 (1.15)	0.04*
	**6 weeks**	2.70 (0.72)	3.07 (1.47)	0.50
	**Friedman test**	0.003*	< 0.001*	
	**Baseline vs. 3 weeks**	0.02*	< 0.001*	
	**Baseline vs. 6 weeks**	0.003*	< 0.001*	
	**3 weeks vs. 6 weeks**	1.00	1.00	
**Head and neck**	**Baseline**	1.02 (0.74)	1.31 (0.91)	0.38
	**3 weeks**	2.38 (1.14)	3.39 (1.52)	0.04*
	**6 weeks**	2.38 (0.86)	3.63 (1.39)	0.02*
	**Friedman test**	0.001*	< 0.001*	
	**Baseline vs. 3 weeks**	0.01*	< 0.001*	
	**Baseline vs. 6 weeks**	0.002*	< 0.001*	
	**3 weeks vs. 6 weeks**	1.00	1.00	
**Total score**	**Baseline**	0.95 (0.50)	0.99 (0.58)	0.79
	**3 weeks**	2.34 (1.43)	3.04 (1.23)	0.04*
	**6 weeks**	2.34 (0.66)	3.04 (1.30)	0.16
	**Friedman test**	0.004*	< 0.001*	
	**Baseline vs. 3 weeks**	0.02*	< 0.001*	
	**Baseline vs. 6 weeks**	0.007*	< 0.001*	
	**3 weeks vs. 6 weeks**	1.00	1.00	

*statistically significant at p value < 0.05

Table 8 represents the oral microbiome results at baseline and after six weeks of therapy. No significant differences between groups were reported at baseline (p > 0.05). The Bacteroidetes phylum was significantly higher in the test group at six weeks (mean (SD) = 62 (13) ×10^− 2^ and 38 (28) ×10^− 2^ in the test and control groups, respectively, p = 0.04). Moreover, the difference from baseline in both the Bacteroidetes and Firmicutes phyla differed significantly between both groups (p = 0.002 and 0.02, respectively). Intragroup comparisons also showed a significant change in the Bacteroidetes phylum in both groups (p = 0.04), and also a significant increase in the Firmicutes phylum in the control group only (p = 0.01). Meanwhile, the Firmicutes to Bacteroidetes ratio was significantly higher in the control group at the six weeks follow-up (mean (SD) = 6.96 (11.24) compared to 1.32 (0.30), p = 0.01), and the difference from baseline also differed significantly between both groups (p = 0.002). The intragroup comparison of Firmicutes to Bacteroidetes ratio differed between baseline and the 6-week follow-up in the control group only (p = 0.01).

**Table 8:** Microbiome results in the two study groups

		Test	Control	P value 1
		Mean (SD)		
**Bacteroidetes**	**Baseline**	55 (19) ×10^− 2^	46 (24) ×10^− 2^	0.32
	**Six weeks**	62 (13) ×10^− 2^	38 (28) ×10^− 2^	0.04*
	**Difference.**	7 (17) ×10^− 2^	-8 (9) ×10^− 2^	0.002*
	**P value 2**	0.04*	0.04*	
**Firmicutes**	**Baseline**	84 (7) ×10^− 2^	81 (11) ×10^− 2^	0.67
	**Six weeks**	88 (9) ×10^− 2^	94 (5) ×10^− 2^	0.06
	**Difference**	4 (6) ×10^− 2^	13 (7) ×10^− 2^	0.02*
	**P value 2**	0.07	0.01*	
**Firmicutes: Bacteroidetes ratio**	**Baseline**	1.73 (0.74)	4.10 (6.85)	0.61
	**Six weeks**	1.32 (0.30)	6.96 (11.24)	0.01*
	**Difference**	-0.41 (0.71)	2.86 (4.46)	0.002*
	**P value 2**	0.11	0.01*	

P value 1: Test compared to control (Mann-Whitney U test)

P value 2: Baseline compared to follow-up (Wilcoxon signed rank test)

*statistically significant at p value <0.05

## Discussion

RIOM is one of the most frequently encountered complications in HNC [71], that is usually associated with significant pain and discomfort, affecting the patient’s QoL [9]. Omega-3 PUFAs have been reported to have vital roles in inflammation reduction and tissue homeostasis recovery in many oral diseases including oral mucositis [72]. Results of the present study demonstrated a significant difference in VAS pain scores between the test and control groups after six weeks. This comes together with a significant reduction in oral mucositis incidence and severity in the test group in both times of assessments. After six weeks, around (59%) of the test group were clinically free from oral mucositis compared to only two patients in the control group. These results highlighted the efficacy of topical Omega-3 nanoemulgel in preventing oral mucositis, mitigating its severity and associated pain. This could be possibly attributed to the previously illustrated potent anti-inflammatory, antioxidant roles and early wound epithelialization ability. No adverse side-effects were observed in both groups, however, the only complaint received was a slight fishy taste associated with topical Omega-3.

Our results are aligned with Hashemipour et al. [40] who reported a significant decrease in oral mucositis severity and duration in patients receiving systemic Omega-3 supplements. Furthermore, our results are also consistent with El Khouli et al. [39] who reported a significant reduction in RAS outbreak and pain level after using ω-3 supplements [39]. Despite the difference in the etiopathogenesis between RAS and RIOM, the study highlights the influence of omega-3 intake on mucosal recovery [72].This can be related to the ability of EPA and DHA, on a cellular level, to maintain epithelial integrity and cell barrier function by preventing disruption in tight junction structure and decreasing cell necrosis [73]. In addition, our results of topical protective action of Omega-3 nanoemulgel are comparable with Basha et al. [28] who demonstrated a significant enhancement in oral mucosal wound healing in rats treated topically by omega-3 compared to its systemic administration.

Although clinician-reported instruments such as the WHO grading system can provide an adequate estimate of the severity of oral mucositis and its associated symptoms, patient reported outcome tools have been increasingly used to measure symptom burden and QoL [64]. In our study, the MDASI-HN was chosen to assess patients’ symptom severity during the study period. MDASI-HN is a brief, validated, comprehensive, self-administered questionnaire that directly assesses HNC symptoms and is closely associated with the severity of RIOM [74, 75].

Our study demonstrated a significant difference in MDASI-HN scores in the head and neck subscale between groups at three and six weeks, while the total scores differed significantly only after three weeks. Patients receiving topical omega-3 nanoemulgel experienced clinically meaningful reduction in mouth sores and reported easier food chewing and swallowing, together with an overall improvement in their daily functioning and QoL compared to the control group. These results are consistent with Barker et al [76] who reported a significant worsening in most of MDASI-HN scores, where difficulty in swallowing, oral dryness and mouth sores were the most clinically deteriorating symptoms experienced by patients, after six weeks of receiving (chemo)radiotherapy in the head and neck region. However, in contrast to our findings, Lopez et al [77] reported no significant differences in any of the MDASI-HN items after six weeks of oral glutamine administration compared to placebo, and this could be related to the non-significant difference in clinically reported outcomes between the two studied groups.

In addition to the known mechanisms of RIOM development and progression, recently, there have been a surge of interest in microbiome dysbiosis and its possible association with oral mucositis [78–81]. ω-3 is currently identified as a major potential hotspot in managing gastrointestinal and oral bacterial dysbiosis [82, 83]. In our study, we mainly focused on dominant bacterial phyla [22]: Bacteroidetes and Firmicutes where Firmicutes/Bacteroidetes ratio could serve as a marker for bacterial dysbiosis [84]. After six weeks, our results have outlined a significant increase in Firmicutes and Firmicutes/Bacteroidetes ratio in the control group compared to the test group. These results are consistent with the Pilchardus Study (2016)which reported a significant decrease in the Firmicutes phylum in both experimental groups, together with a decrease in Firmicutes/Bacteroidetes ratio in the Omega-3 group [85]. This was also aligned with Yu et al. who reported a reduction in the Firmicutes phylum in mice fed by ω-3 rich fish oil for 15 days [86]. Additionally, Fu et al. illustrated the inherent ability of ω-3 supplementations in altering the abundance and diversity of gut microbes, specifically influencing Firmicutes/Bacteroidetes ratio in many diseases such as Obesity and inflammatory bowel disease [83].

The study has several strengths, to the best of our knowledge, this is the first clinical trial to test the efficacy of topical Omega-3 nanoemulgel in preventing RIOM. We depended on clinical examination to assess the oral mucositis severity and complemented our assessment by measuring patient-reported outcomes to comprehensively capture the efficacy of topical omega-3 in prevention of RIOM and its associated symptoms. We also collected oral swabs to assess the role of Omega-3 in regulation of oral microbial dysbiosis. Our study, thus, fills a knowledge gap by providing evidence about the effect of topical Omega-3 nanoemulgel in prevention of RIOM.

However, the study had some limitations including the short-term follow up, so further clinical trials with larger sample size and longer follow-ups that can assess OM after several radiation therapy-cycles are still needed. Also, the current study included a heterogenous group of head and neck tumours that required different radiation doses for treatment, thus, more clinical trials are needed with standardization of the type of head and neck cancer to ensure a more homogenous sample of patients. Also, trials comparing the efficacy of topical and systemic Omega-3 supplements on mucositis prevention are needed to further study the role of Omega-3 on modulation of oral microbiota, including more phyla and species. More studies are encouraged to determine and adjust the exact dosage of Omega-3 nanoemulgel in different oral diseases.

## Conclusions

The current study demonstrated a beneficial effect of topical use of ω-3 nanoemulgel in RIOM prevention, decreasing the associated pain intensity and improving the overall QoL of HNC patients. Moreover, in addition to its potent anti-inflammatory and antioxidant properties, our study suggests a potential role of Omega-3 in influencing oral microbial dysbiosis that could directly or indirectly help in RIOM amelioration.

## Data Availability

All data included in this study are available from the corresponding author upon request.

## List of abbreviations

OM: Oral mucositis
HNC: Head and neck cancer
ω-3: Omega-3
PUFAs: Polyunsaturated fatty acids
WHO: World Health Organization
VAS: Visual Analogue Scale
MDASI-HN: MD-Anderson Symptom Inventory for Head and Neck cancer
RIOM: Radiation-induced oral mucositis
QoL: Quality of life
ROS: Reactive oxygen species
NF-κB: Nuclear factor kappa B
TNF-α: Tumor necrosis factor alpha,
IL-1β: Interleukin-1 beta
IL-6: Interleukin6
EPA: Eicosapentaenoic acid
DHA: Docosahexaenoic acid
SPMs: Pro-resolving mediators
RAS: Recurrent aphthous stomatitis
NE: Nanoemulsion

## Footnotes

1 Manufactured by Medical Union Pharmaceuticals, 6th District-Nasr City- Cairo-A.R.E.

2 Manufactured by: Misr Co. For Pharm. Ind. S.A.E. - Egypt.

3 Manufactured by Amoun Pharmaceutical Company El Obour city – Cairo – Egypt.

4 Manufactured by Amoun Pharmaceutical Company El Obour city – Cairo – Egypt.
